# Open‐source data reveal how collections‐based fungal diversity is sensitive to global change

**DOI:** 10.1002/aps3.1227

**Published:** 2019-03-12

**Authors:** Carrie Andrew, Ulf Büntgen, Simon Egli, Beatrice Senn‐Irlet, John‐Arvid Grytnes, Jacob Heilmann‐Clausen, Lynne Boddy, Claus Bässler, Alan C. Gange, Einar Heegaard, Klaus Høiland, Paul M. Kirk, Irmgard Krisai‐Greilhüber, Thomas W. Kuyper, Håvard Kauserud

**Affiliations:** ^1^ Swiss Federal Research Institute WSL CH‐8903 Birmensdorf Switzerland; ^2^ Section for Genetics and Evolutionary Biology (EVOGENE) University of Oslo Blindernveien 31 0316 Oslo Norway; ^3^ Department of Geography University of Cambridge CB2 3EN Cambridge United Kingom; ^4^ Global Change Research Centre and Masaryk University 613 00 Brno Czech Republic; ^5^ Department of Biological Sciences University of Bergen P.O. Box 7803 N‐5020 Bergen Norway; ^6^ Centre for Macroecology, Evolution and Climate Natural History Museum of Denmark University of Copenhagen DK‐2100 Copenhagen Denmark; ^7^ School of Biosciences Cardiff University Museum Avenue Cardiff CF10 3AX United Kingdom; ^8^ Bavarian Forest National Park Freyunger Str. 2 94481 Grafenau Germany; ^9^ Chair for Terrestrial Ecology Technical University of Munich Hans‐Carl‐von‐Carlowitz‐Platz 2 85354 Freising Germany; ^10^ School of Biological Sciences Royal Holloway University of London Egham Surrey TW20 0EX United Kingdom; ^11^ Forestry and Forest Resources Norwegian Institute of Bioeconomy Research Fanaflaten 4 N‐5244 Fana Norway; ^12^ Mycology Section Jodrell Laboratory Royal Botanic Garden Kew, Richmond Surrey TW9 3DS United Kingdom; ^13^ Department of Botany and Biodiversity Research University of Vienna 1030 Vienna Austria; ^14^ Department of Soil Quality Wageningen University P.O. Box 47 6700 AA Wageningen The Netherlands

**Keywords:** collections data, diversity, fungi, macroecology, open‐source, phenology records

## Abstract

**Premise of the Study:**

Fungal diversity (richness) trends at large scales are in urgent need of investigation, especially through novel situations that combine long‐term observational with environmental and remotely sensed open‐source data.

**Methods:**

We modeled fungal richness, with collections‐based records of saprotrophic (decaying) and ectomycorrhizal (plant mutualistic) fungi, using an array of environmental variables across geographical gradients from northern to central Europe. Temporal differences in covariables granted insight into the impacts of the shorter‐ versus longer‐term environment on fungal richness.

**Results:**

Fungal richness varied significantly across different land‐use types, with highest richness in forests and lowest in urban areas. Latitudinal trends supported a unimodal pattern in diversity across Europe. Temperature, both annual mean and range, was positively correlated with richness, indicating the importance of seasonality in increasing richness amounts. Precipitation seasonality notably affected saprotrophic fungal diversity (a unimodal relationship), as did daily precipitation of the collection day (negatively correlated). Ectomycorrhizal fungal richness differed from that of saprotrophs by being positively associated with tree species richness.

**Discussion:**

Our results demonstrate that fungal richness is strongly correlated with land use and climate conditions, especially concerning seasonality, and that ongoing global change processes will affect fungal richness patterns at large scales.

Collections databases formed through museum specimens and curated citizen science data (inclusive of individual and group “amateur” collections and surveys) are ideal for macroecology research, relating the presence of organisms to global change and, especially, past to present impacts of climate (Lavoie, 2013; Wen et al., 2015; Andrew et al., 2017; Willis et al., 2017). Patterns and processes governing the biogeographical distributions of organisms in their natural environments can be elucidated across geographical gradients at previously unprecedented scales (Andrew et al., 2018a, b). The environmental roles shaping fungal diversity are open for investigation, especially within a backdrop of global change (Fisher et al., 2012; Pärtel et al., 2016; Soudzilovskaia et al., 2017; Titeux et al., 2017; Mucha et al., 2018).

With very few exceptions, there have been no large‐scale quantitative studies on this theme across comprehensive spatial gradients. The research of Tedersoo et al. (2014) and Davison et al. (2015) have highlighted environmental structuring of continental‐scale fungal diversity, indicating greater mycorrhizal fungal diversity at temperate and boreal localities than the tropics and raising questions of how fungal diversity patterns compare to those of other organisms (Peay, 2014; Peay et al., 2017). However, such studies included a scattering of DNA‐based sample locations, with no more than grams‐worth of soils analyzed per sample. The trends indicated in these studies therefore require verification via different data sources and across more complete spatial gradients.

Fungal records data (inclusive of physical, survey, and curated citizen science data collections, i.e., collections‐based) are an ideal complement to molecular‐based data studies, as they include a huge number of survey locations with high records density, accumulated over many years (Andrew et al., 2017). Fungi are ubiquitous in terrestrial systems. Those that produce macroscopic fruit bodies (sporocarps), which are the sexually reproductive structures, are the most visible and easily recorded. Collections records are based almost entirely on fruit body sightings (including specimen records) and, like all other approaches, cannot target all fungal species (even molecular methodologies produce biases and sampling errors; e.g., Bellemain et al., 2010; Hibbett et al., 2016; Tedersoo and Lindahl, 2016). A substantial amount of saprotrophic (decaying) and ectomycorrhizal (plant mutualistic) species, however, produce macroscopic fruit bodies (Boddy et al., 2014; Runnel et al., 2015). Hence, when collected over time (i.e., decades) and space (i.e., across regions), they can provide powerful data for analyzing macroecological trends for fungi, at unprecedented spatial scales with more complete environmental gradients (Halme et al., 2012; Andrew et al., 2017).

While a global change context to understanding fungal richness patterns will assist our understanding of potential future responses (i.e., which environmental components should be targeted in projection or experimental approaches; Mair et al., 2018; Mucha et al., 2018), technical issues concerning the spatiotemporal availability of associated, open‐access environmental (e.g., climate and land‐use) data can hamper inferences and analyses. The open‐access explanatory variables used to assess environmental gradients differ (in source[s], format[s], and resolution[s]), restricting what questions can be ecologically asked, despite these being the only environmental data available for inquiry at such large spatial scales that extend, minimally, decades into the past. For example, environmental data may either lack representation across the entire area covered or, more often, only be available at varying coarser spatial resolutions than the collections data (ca. 1–20 km^2^ most often). Conducting analyses at coarser grid levels (e.g., 50 km^2^) is often more appropriate for large‐scale analyses, and simultaneously reduces coexisting spatiotemporal autocorrelation issues (that can be further attended to during modeling).

Often, only single, static time‐period measures are available to link up current conditions with the biological data; these measures lack the dynamism (daily to annual variability) that is key to our understanding, given that we are accelerating climate change and grossly modifying the landscape (Holyoak and Heath, 2016). For example, urbanization and forest management each impact biodiversity (Paillet et al., 2010; Dvořák et al., 2017), with interactions between land‐use change and climate (Mair et al., 2018) as well as tree species diversity (Spake et al., 2016). Likewise, climate affects assemblages of fungi, which consequently impacts phenological patterns (Talbot et al., 2014; Andrew et al., 2018a, b). Potentially cascading effects include those to diversity, dispersal, and composition (Titeux et al., 2017). Thus, to aptly put the *change* in global change research, we must employ novel techniques to quantify temporal movement linking organisms to their environment(s) at the time of recording—and at scales as appropriate as possible for capturing their responses.

Here we examined how large‐scale environmental gradients have structured contemporary to recent historical fungal diversity patterns. We utilized a novel data source (the ClimFun meta‐database; Andrew et al., 2017), connected to an array of environmental covariates with as much spatiotemporal resolution as possible, across a large geographical gradient spanning central to northern Europe. Our objectives were multi‐faceted, integrating an overall, macroecological, and global change context with applied goals regarding the consequences of temporal accuracy in environmental covariates:

1. Our ultimate objective was to understand how global change (using data from 1970 to 2010), especially land use and climate change, can impact the diversity of fungi at broad spatiotemporal scales.
2. Via generalized additive mixed modeling, we investigated how geographically based environmental patterns structured fungal richness. As land use classifies the habitat that fungi are found within, we first examined its influence on fungal richness. Next, available environmental attributes, such as climate‐related variables, tree species richness, and pollution, were modeled with respect to their impacts on fungal richness, while controlling for differences between land‐use types. Finally, based on earlier studies (Tedersoo et al., 2014), we investigated the extent that collections‐based data confirm that fungal richness is nonlinearly correlated with latitude (i.e., greater diversity in temperate to boreal regions).
3. Throughout the study, we explored how temporal accuracy in environmental data affected the inferences of global change impacts. We compared, as possible, the predictive capacity of data originating as either temporally static (a single time unit) or dynamic (daily to annual values), expecting greater accuracy from the latter, especially for land‐use and climate data (temperature, precipitation). Being linked to the collections data at the finest temporal resolution possible, the most precise conditions possible on the day of recording were established (collection day conditions), capturing global change impacts in situ.

## METHODS

Fungal data from a collections‐based “meta‐database” of fruit body records, with millions of records from the 18th century forward, originated from independent national‐scale data repositories in Europe and included physical, survey, and curated citizen science data collections (Andrew et al., 2017). All available national‐scale and regional‐scale (i.e., semi‐national) data were included during preparations, although ultimately data from Estonia (a national source) and Sweden (a semi‐national source) were excluded because too few records were available (after preparation). The timespan was limited to 1970–2010 because the majority of records were within this range and because it reduced collection bias of earlier years. The taxonomy was limited to the major mushroom orders (toadstools, bracket fungi) in the Agaricomycotina (removing the Cystofilobasidiales and Trichosporonales). Careful and extensive formatting and filtering assured the quality of the data at multiple stages: when the fungal records were originally combined as well as during the initial linkage of temporally static metadata (Andrew et al., 2017), and after the addition of newly available and/or temporally dynamic environmental data novel to the research presented here.

All open‐access environmental data, available across all countries within the meta‐database, were linked up to individual records at the finest spatiotemporal resolutions possible. Nine data sources, with a total of 34 variables, spanned six categories of ecological relevance (Table 1): climate (WorldClim and E‐OBS), land‐use type (CORINE Land Cover [CLC] 2006 and Integrated Science Assessment Model–Historical Database of the Global Environment [ISAM‐HYDE]), normalized difference vegetation index (NDVI; a proxy for primary production), nitrogen deposition (NH_x_, NO_y_), tree species composition and richness, and soil organic carbon (Table 1). As much as possible, variables with temporally dynamic (daily, monthly, annual values) information were included, being linked up to collection records at the finest temporal resolution as possible.

**Table 1:** The fungal and open‐access environmental covariates used to investigate fungal richness patterns in Europe.^a^^,^ b

Covariate	Temporal resolution (finest)	Approx. spatial resolution (finest)	Data source	Reference
Fungal richness	Dynamic (daily)	<1× 1 km	ClimFun	Andrew et al., 2017
Tree species richness	Static (single point)	20 × 20 km	EU‐Forest	Mauri et al., 2017
Climate (19 variables)	Static (single point)	1 × 1 km	WorldClim	Hijmans et al., 2005
Climate (2 variables)	Dynamic (daily)	20 × 20 km	E‐OBS	Haylock et al., 2008
NDVI (mean, max.)	Static (single point)	10 × 10 km	ECOCAST	Pinzon and Tucker, 2014
NDVI (mean, max.)	Dynamic (annual)	10 × 10 km	ECOCAST	Pinzon and Tucker, 2014
Soil organic carbon (%)	Static (single point)	1 × 1 km	JRC‐ESDAC	Jones et al., 2005
Land use (3 levels)	Static (single point)	1 × 1 km	CLC (2006)	www.eea.europa.eu
Land use (main)	Dynamic (annual)	20 × 20 km	ISAM‐HYDE	Meiyappan and Jain, 2012
N dep. (NH_x_, NO_y_)	Dynamic (monthly)	20 × 20 km	GHG Europe	europe-fluxdata.eu/ghg-europe

Note

NDVI = normalized difference vegetation index.

a The explanatory variables were available as either temporally static (one time point) or dynamic (multiple time points) values. They were linked up with the collections data as precisely as possible, and then the means, minima, and/or maxima were calculated across 1970–2010 in 50 × 50‐km grids for analyses.

b See Appendices 5, 7, S8, and S11 for collinearity analyses and geographical distributions of the covariates.

The fungal species were assigned nutritional mode (fungal guild) status to help separate them into ecologically relevant groups. Expert opinions were augmented with information from the FUNGuild database to fill gaps when possible (Nguyen et al., 2016; accessed on 14 March 2018). Analyses were limited to two main groups: saprotrophic (decaying) and ectomycorrhizal (plant mutualistic) fungi, which comprised the majority of species and data records (Appendix 1). There were more saprotrophic fungal species than ectomycorrhizal, but the distribution in the amounts of records per species was similar between the two groups (Appendix 2).

Data in 20‐ and 50‐km^2^ grids were compared due to concern for greater spatial bias at lower resolutions (Geldmann et al., 2016; Panchen et al., 2019). The 50‐km^2^ resolution was chosen for all analyses. Richness patterns, however, were similar across both grid resolutions. To further minimize bias, each grid was required to contain a minimal number of records per grid (100–500), with a minimum of 350 and 500 records subsequently selected for further comparison; although patterns were similar, a minimum of 500 records was found to most reduce bias and was selected for the final analyses (Appendix 3). Species with five or fewer occurrences were removed from the data, as approximately half of these were taxonomic discrepancies (J.H.‐C., personal communication). At such broad spatial scales, rare species may have a smaller impact than at more localized scales (Jetz and Rahbek, 2002; Heegaard et al., 2013). Fungal species per grid were rarefied to the minimum records allowed (i.e., 500) to create richness values.

Tree species richness was calculated in a similar way to fungal richness, in terms of all tree species as well as ectomycorrhizal tree species (e.g., those known to be symbiotic with ectomycorrhizal fungi versus those that are not). The latter were classified based on Hempel et al. (2013), Bueno et al. (2017), and Gerz et al. (2018). Gridded total tree species were connected to the gridded fungal data for saprotrophic and ectomycorrhizal fungal groups. Richness patterns matched the data source as well as established European patterns for tree diversity (Mauri et al., 2017; Appendix 4).

Variable selection occurred in three processing stages. In the first, all variables (Table 1) were compared to select those with the lowest pairwise Pearson correlation coefficients (below a threshold of 0.60; Dormann et al., 2013). Additionally, variance inflation factor (VIF) analyses excluded covariates with values ≥5 (a conservative threshold) and/or ≥10 (a less conservative threshold) (Zuur et al., 2009). For pairwise coefficient values and geographical distributions of all variables, see Appendices 5, 6, 7, 8. In the second stage of variable selection, for each variable group (e.g., temperature and/or precipitation values for mean annual, seasonal minima, maxima, and ranges; precipitation‐linked temperature values), one covariate was identified during model selection procedures as being most influential to either saprotrophic or ectomycorrhizal fungal diversity. In the final stage of selection, only those covariates statistically significant in the final models were used for analyses, and a final check of correlation coefficients and VIF was conducted (see below). Variables were scaled and centered for direct comparisons of impacts between them, although results were similar to non‐scaled versions.

Land‐type influences on diversity were investigated prior to modeling environmental impacts, as it was hypothesized that diversity would be strongly influenced by land type. Analysis of variance (ANOVA) and Tukey's honest significant difference (HSD) were used to differentiate statistically significant richness values between land‐use types, focusing on the finest resolution in the temporally static CLC data and on the dominant land classification for the temporally dynamic ISAM‐HYDE data (Appendices 9, 10, [Link], [Link], [Link], [Link], [Link]). The latter, by changing with time, should better represent land‐use type related to where the fungi were recorded. Classifications with low sample sizes and/or that were aquatic (i.e., non‐terrestrial water associations) were removed. Assumptions of normal distributions and homogeneous variances were required. The dynamic land‐cover associations with richness are presented in the Results section, and results from CLC data are available in Appendices 9, 10, and [Link], [Link], [Link], [Link], [Link]. Because sample size was very low for some land‐use types, it was not possible to include interactive impacts between land‐use type and climate.

Generalized additive mixed model (GAMM) regression analyses were utilized to model predicted richness in Europe (Appendices [Link], [Link], [Link], [Link], [Link], [Link], [Link]). Parallel model selection processing occurred for saprotrophic and ectomycorrhizal fungal diversity. Both forward and backward selection procedures were used to identify influential explanatory variables while verifying the robustness of the results (Zuur et al., 2009). A consensus model was then built that combined the covariates selected between the two procedures (Appendix S5). Earlier preliminary analyses compared the selected explanatory variables in models built from linear, linear mixed, generalized linear, and generalized additive models; results were similar across these models. A Gaussian distribution was used with the scaled variables, although the application of a quasi‐Poisson distribution with non‐scaled variables did not impact the model results or fits. Explanatory variables were modeled with splines to allow non‐linear trends, with knot values set at 10, but decreasing to fit the models successfully; this reduction had little impact on the overall results and covariate significances. The interaction of easting and northing was always retained at a specification of *k* = 15 to allow greater non‐linearity for accurate geographical modeling. Land type (ISAM‐HYDE) was included as a random effect. To account for unequal number of records, in addition to the previously explained data preparations, the natural log of the records amounts per grid were included in the model as an offset and weight specification. Spatial autocorrelation was further reduced by including a correlation component (corExp). Model fits were determined via the Akaike information criterion (lowest value), *R* ^2^ (highest value), and spatial autocorrelation assessed via variograms and bubble plots (Zuur et al., 2009). A final check of the selected explanatory variables verified the lowest possible collinearities, with deviations only in terms of geographical relationships (Appendices [Link], [Link]). All data preparations and analyses were conducted in R version 3.2.2 (R Core Team, 2015) with the packages: *corrplot* (correlations), *gstat* (autocorrelation), *maps* (spatial), *mgcv* (regression), *rgdal* (geospatial), *sp* (spatial), *stats* (regression), *stringr* (data formatting), *vegan* (rarefaction), and *usdm* (VIF).

## RESULTS

**Figure 1:**
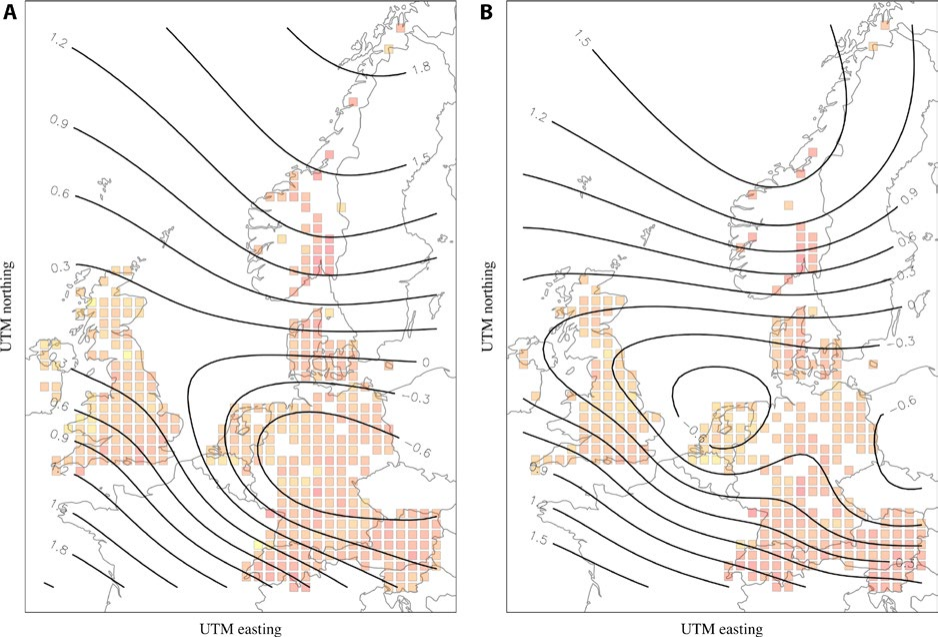
The geographical patterns of predicted richness (isolines) for saprotrophic (A) and ectomycorrhizal (B) fungi. The background grids are shaded by values of the actual, rarefied richness, with lower values in yellow grading to higher values in red. Richness and Universal Transverse Mercator coordinate system values are presented as scaled and centered versions.

In general, richness patterns followed a unimodal distribution across the latitudinal gradient range. Higher predicted values occurred in northern and central localities, with fewer longitudinal impacts (Fig. 1; Appendices [Link], [Link]). Land‐use categories could, in part, explain these trends (Appendices [Link], [Link]), especially in terms of the impacts of non‐forested and urban systems on geographical richness patterns.

### Land use and fungal richness

**Figure 2:**
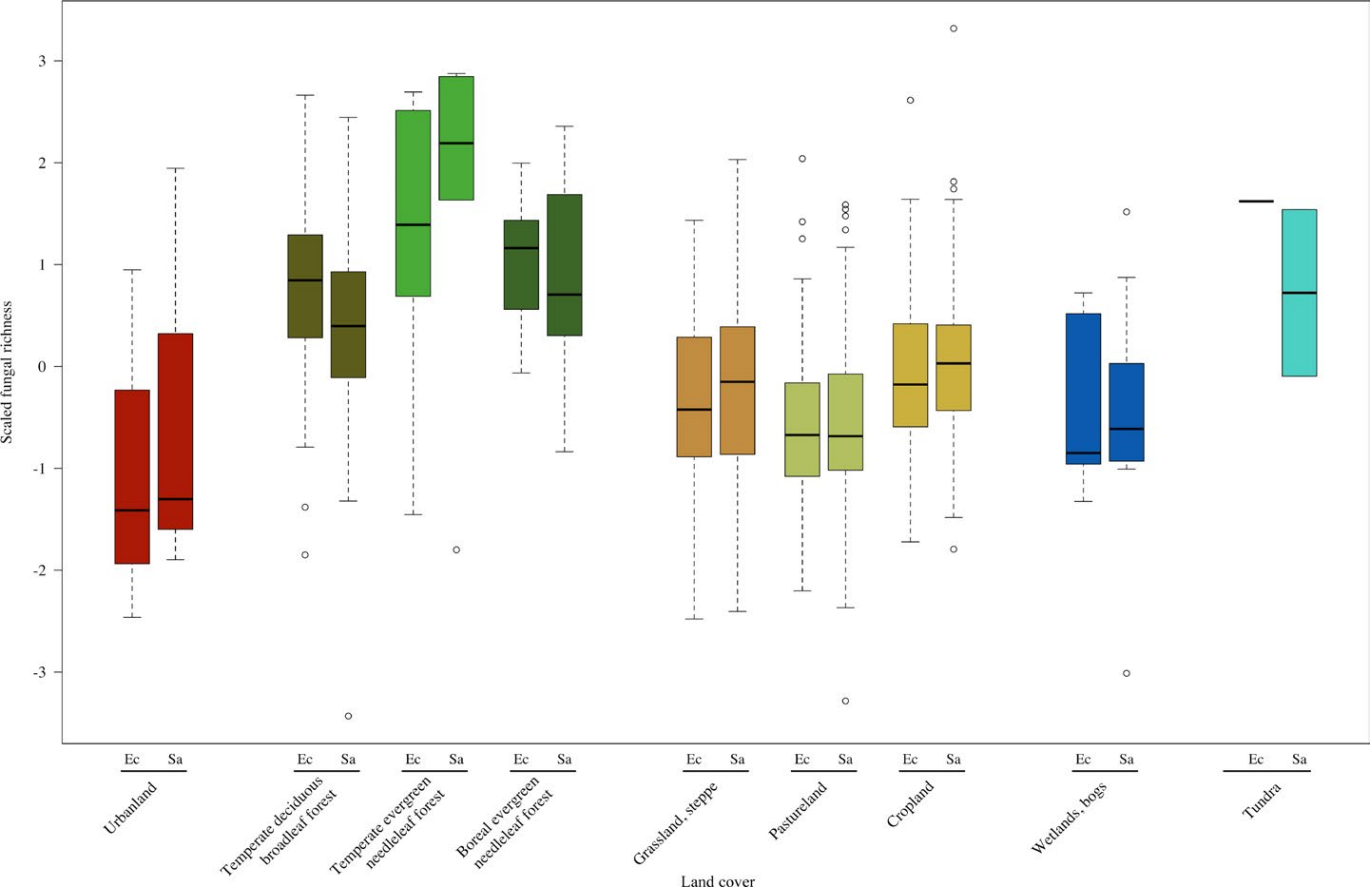
Land‐use type impacts on richness of ectomycorrhizal (Ec) and saprotrophic (Sa) fungi, with scaled and centered values for direct comparisons, according to the dynamic land‐cover covariate ISAM‐HYDE. Values were calculated by classifying each 50 × 50‐km grid based on the highest amount of land type as associated with the fungal collections data by spatial and temporal points.

Land‐use type impacted the richness of both saprotrophic and ectomycorrhizal fungi (Fig. 2, Appendix 9). Forested systems had greater fungal richness than grassland, pasture, and agricultural lands, and markedly greater richness for ectomycorrhizal than saprotrophic fungi (Appendices [Link], [Link], [Link], [Link], [Link]). Urban diversity means were lower than in non‐forested systems, although all classifications had substantial variability (Fig. 2). Notably, more pairwise comparisons between urban land and the three forested types were statistically significant for ectomycorrhizal than for saprotrophic fungi (Appendices [Link], [Link]). Greatest mean diversity of fungi was found within temperate evergreen needleleaf (coniferous) forests, with more similar values between temperate deciduous broadleaf forests and boreal evergreen needleleaf (coniferous) forests (Fig. 2). These latter two forest types were also noteworthy in that they were the exceptions for what were otherwise trends of greater mean richness by saprotrophic than ectomycorrhizal fungi for a given land‐use type. The effects on mean values were very similar between the presented, dynamic (ISAM‐HYDE) land‐use classifications and the supplemental, static (CLC) land‐use classifications results (Fig. 2, Appendix 10).

### Climate and fungal richness

**Figure 3:**
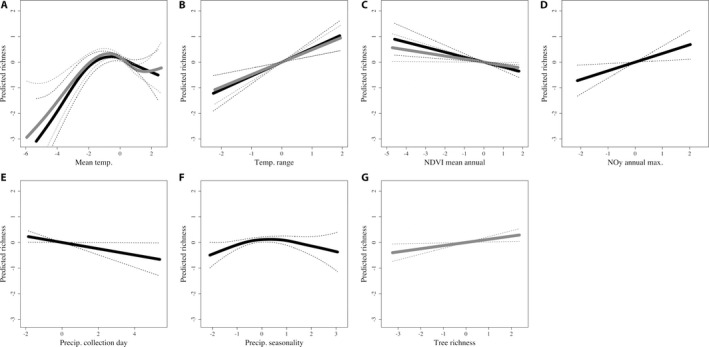
For saprotrophic (black) and ectomycorrhizal (gray) fungi, the modeled predictions of statistically significant (A–C) and marginally significant (D–G) environmental impacts on fungal richness. Main effects are shown as solid lines; 95% confidence intervals are shown as dotted lines. Inclusion of saprotrophic or ectomycorrhizal fungal diversity predictors is contingent on the significance of the covariate in the final model. All values are provided in scaled and centered formats.

Climate was the prevailing force structuring fungal diversity patterns in central to northern Europe, followed by primary productivity, nitrogen deposition, and seasonally related precipitation measures (Table 2, Fig. 3, Appendix S12). Richness of both saprotrophic and ectomycorrhizal fungi was most strongly influenced by two static temperature values (mean annual temperature and annual temperature range [all *P* values ≤ 0.01; positive correlations]) and mean annual NDVI values (*P* ≤ 0.01 and 0.04, respectively; negative correlations).

**Table 2:** ANOVA tables for the generalized additive mixed model (GAMM) regressions predicting richness of saprotrophic and ectomycorrhizal fungi.^a^

Explanatory variable	edf	Ref.df	*F*	*P* value
Saprotrophic richness				
Geogr., easting : northing (UTM)	9.79	9.79	4.91	1.360E‐06
Temp., mean annual (BIO1)	3.39	3.39	7.03	7.100E‐05
Temp., annual range (BIO7)	1.00	1.00	12.37	4.890E‐04
NDVI, mean annual	1.00	1.00	8.30	0.004
NO_y_, annual max.	1.00	1.00	5.73	0.017
Precip., collection day	1.00	1.00	4.26	0.040
Precip., seasonality (BIO15)	2.42	2.42	3.21	0.043
Ectomycorrhizal richness				
Geogr., easting : northing (UTM)	10.93	10.93	7.67	8.110E‐12
Temp., mean annual (BIO1)	4.04	4.04	6.79	3.210E‐05
Temp., annual range (BIO7)	1.00	1.00	14.67	1.550E‐04
Tree richness, ectomycorrhizal spp.	1.00	1.00	5.53	0.019
NDVI, mean annual	1.00	1.00	4.33	0.038

Note

NDVI = normalized difference vegetation index; UTM = Universal Transverse Mercator coordinate system.

a See Methods and Appendices 5, 6, 7, 8, [Link], [Link], [Link], [Link], [Link], [Link], [Link] for further information regarding model specifications and selection processing.

Whereas temperature and NDVI helped explain richness for both saprotrophic and ectomycorrhizal fungi, there were further, separate explanatory variables that were important for each nutritional mode. Saprotrophic fungal diversity was influenced by annual maximum NO_y_ (*P* ≤ 0.02; positive correlation), precipitation averaged from the collection day (*P* ≤ 0.04; negative correlation), and seasonality of overall precipitation (*P* ≤ 0.04; unimodal; Fig. 2, Appendix S12). This contrasts with ectomycorrhizal fungi, for which the richness of ectomycorrhizal trees was the only additional, statistically significant explanatory variable (*P* ≤ 0.02; positive correlation; Fig. 2, Appendix S13).

### Static versus dynamic variables

Given the data available, temporally static (one time point) and dynamic (daily, monthly, annual) variables were most comparable for temperature and precipitation (Appendices 5, 6, 7, 8). Precipitation averaged from the collection days correlated to the overall mean precipitation (WorldClim, variable BIO12) within a range of 0.76–0.77. Precipitation at that scale, even when averaged across 1970–2010, more accurately captured rainfall along coastal Europe as opposed to overall values (Appendices 6, 8). The correlation between temperature of the collection day versus mean annual values was even less, ranging from 0.49–0.57 (ectomycorrhizal and saprotrophic, respectively). Less correlation between static and dynamic temperatures likely occurred because the minimal temperature of the former was essentially lacking from the latter, especially for northern locations, thus modifying the overall values to capture fruiting season temperatures (Appendices 5, 8).

## DISCUSSION

The unimodal trend in large‐scale diversity related to latitude continues to gather support for fungi, with our findings aligning with those of earlier research across a variety of study locations and data sources (Shi et al., 2014; Tedersoo et al., 2014). It is especially interesting that fungi apparently do not align with diversity patterns of other organisms (Colwell et al., 2004; Hillebrand, 2004; Peay et al., 2017), given their biotic interactions with, for example, trees (Fig. 2). Although we cannot ascertain the exact reason(s), we do recommend further investigating the relationships to land‐use type and then, especially, climate and its variance related to seasonality.

### Land use and fungal richness

The higher predicted richness values in northern and south‐central European localities (Fig. 1), with reduced predictions in the areas in between, are at least partly due to a few important distinctions along the gradient (Appendices [Link], [Link]). First, urban locations were found, for our analyses utilizing a 50‐km^2^ grid, exclusively at lower latitudes. Consistent with previous research (Schmidt et al., 2017), mean diversity in urban systems was reduced for both fungal nutritional modes (Fig. 2), with greater negative impacts to ectomycorrhizal fungal diversity in urban systems compared to forests (Appendices [Link], [Link]). These findings correlate well with the known effects of urbanization on tree diversity at the landscape scale (Vallet et al., 2008). Non‐forested grassland and agricultural systems dominated latitudinally central localities, with differences between grassland and forest diversity matching those of other studies (Divíšek and Chytrý, 2018), but on a much broader scale. These results independently verify that collectors are not biasing, as much as they are knowledgeable of, areas with high richness (i.e., Geldmann et al., 2016). With respect to the large‐scale unimodal fungal diversity trends we have found, the geographical locations of urban and non‐forested land types appear to help explain this trend.

Temperate evergreen forests contained the highest levels of fungal diversity (Fig. 2). These results support those of other researchers, who have similarly found temperate forests to contain greater fungal diversity, as investigated in a Chinese latitudinal study (Shi et al., 2014) as well as a geographically extensive (but lower‐replication) molecular‐based study (Tedersoo et al., 2014). It is important to note that we can refine this trend and pinpoint the importance of temperate evergreen (coniferous) forests, compared to temperate deciduous broadleaf forests, as harbingers of high fungal richness. Because management can impact forests beyond what we have demonstrated here (Paillet et al., 2010; Spake et al., 2016; Dvořák et al., 2017; Mair et al., 2018), studies regarding the interactions between management and forest types can clarify an area that is of clear importance for fungal diversity.

A separate issue regarding impact of land‐use type on richness was reflected in what could be low sample sizes within certain areas. For example, there was an indication of high diversity in tundra systems (Fig. 2), but the minimum record amount specifications (500 required) within each of what were rather coarse‐scale grid resolutions (50 km^2^) resulted in too few sample points to conclusively demonstrate this trend. While inevitable for these analyses, we recommend further investigation at smaller spatial resolutions, if it is possible to remedy existing sampling biases (that prohibited us here). More localized comparative studies could also provide clarification (Gange et al., 2018).

Ultimately, the main reason for differences in fungal communities among forest systems relates to the composition and diversity of trees in them, especially deciduous vs. evergreen, and broadleaf vs. conifer traits. We revealed a positive relationship between fungal (particularly, ectomycorrhizal) and tree richness (Figs. 1, 2), as has also been found for smaller‐scale or coarser‐resolution studies (Shi et al., 2014; Spake et al., 2016; Ordynets et al., 2018). However, urban systems contained high tree diversities (Appendices [Link], [Link]), but this clearly did not correspond to increased fungal diversities (Fig. 2). Similarly, tree species richness could not explain the high‐latitude fungal richness (Fig. 1, Appendix S13), indicating that latitudinally based trends in the environment cannot capture the distribution of fungal richness as appropriately as climate and knowledge of land use can.

### Climate and fungal richness

Plant–mycorrhizal type distributions vary across Europe in relation to mean temperature values (Bueno et al., 2017; García de León et al., 2018), and this corresponds extremely well with our predictions of the effect of environmental attributes on fungal richness. Temperature, both annual mean and range, was positively correlated with richness, indicating that warm environments promoted diversity, as did seasonally extreme environments (seasonal fluctuations in temperature and/or precipitation) (Fig. 3). This further relates to the mid‐latitude and forest system trends just discussed, but emphasizes the importance of climate drivers directly on fungal richness, as well as indirectly via effects on the richness of forest trees (e.g., Andrew et al., 2018a). This distinction not only emphasizes the importance of temperature related to seasonality, but also explains how northern regions, with lower mean annual temperatures, can support higher fungal diversity.

In contrast to temperature, daily precipitation that had been linked to the fungal record (i.e., collection day precipitation, the temporally dynamic variable) was a better predictor of saprotrophic fungal richness (Table 2, Fig. 2, Appendix S14). Seasonality is likely the reason for the significance of collection day precipitation, as its patterns better captured coastal highs of western Europe (Appendix 6). That a precipitation signal was picked up contrasts with previous research on phenology (Andrew et al., 2018a) and assemblages (Andrew et al., 2018b), and demonstrates the power of dynamic covariates to enhance our understanding of the effect of global change on fungi.

NO_y_ deposition levels were highest in urban, cropland, and temperate deciduous forests (Appendices 6, 8, [Link], [Link]), with effects on the latter helping to explain the positive correlation between NO_y_ and saprotrophic fungal richness (Figs. 1, 2). Again, this emphasizes the importance of land‐use type to understanding environmental impacts on diversity (van Strien et al., 2018). In line with this explanation, van der Linde et al. (2018) found negative consequences by nitrogen deposition on ectomycorrhizal fungal taxonomic diversity to be greatest with those fungi associated with conifer trees in boreal regions. The authors argued that northern conifer‐associating fungi are more susceptible to nitrogen pollution, being exposed less often to critical levels, than consistently exposed deciduous‐associating fungi of more central to southern regions. In areas of higher exposure, abatement is reducing diversity losses (van Strien et al., 2018). Because the nitrogen data utilized in this study follow large‐scale deposition patterns, the relative impact of an increase in more pristine northern regions was not captured by the data or the correlations. Hence, we caution against assuming a positive correlation between certain forms of nitrogen (NO_y_) and saprotrophic fungal diversity without understanding the landscape context and/or incorporating relative‐impact covariates of pollution.

### Static versus dynamic variables, and future global change consequences

We found reasonable support for increased accuracy in modeling fungal diversity via temporally dynamic covariates, which we expected as it is well established that species‐specific fruiting is cued by episodic changes in, especially, precipitation and temperature (e.g., Andrew et al., 2018a; Gange et al., 2018). Environmental variables that fluctuated on finer spatial scales and whose relationships to fungal biology acted on smaller scales than overall means (e.g., daily temperature and precipitation) were especially strengthened by the inclusion of a dynamic covariate for analyses. However, further studies concerning biological aspects of organisms that are more tightly linked to timing of fruiting, as opposed to mean diversity measures across decades, will likely benefit more from temporally dynamic measures. Richness itself is also not the only proxy for diversity; beta‐diversity (turnover and homogeneity) and temporal‐based changes would be optimal next steps for research, especially along gradients such as these data comprise.

Major differentiating features of forests that can help predict fungal diversity patterns (i.e., deciduous vs. evergreen and broadleaf vs. needleleaf) were only captured via the temporally dynamic ISAM‐HYDE classifications. The distinction of diversity by forest type (Appendices [Link], [Link]) suggests that the tree traits that may best influence fungal richness patterns are related to the leaves. Leaf traits correspond to both geographical biomes (i.e., climate) as well as to distinct differences in carbon cycling and transference in the trees (Bueno et al., 2017; García de León et al., 2018). It is important to note that needleleaf evergreen forest conversion may cause the greatest loss in fungal richness, although combining these results with knowledge of biotic homogeneity between land types would further elucidate land‐use change impacts (Pärtel et al., 2016). When combined with globalization‐mediated dispersion of pathogens (e.g., Fisher et al., 2012), we can expect rates of species loss to become compounded from land‐use change to urban and non‐forested systems.

Our most important goal was to understand the consequences of global change on large‐scale fungal diversity patterns. The utility of real‐time, dynamic environmental covariates is high for collections‐based data, which extend from decades in the past to contemporary times. The global change component is implicit in the biological response(s) measured; i.e., fungal richness patterns were best predicted by increasing temperature as well as by seasonal extremes in temperature and precipitation. In Europe, mean annual temperature is predicted to continue increasing while mean annual precipitation will, more or less, increase in northern Europe but decrease in southern Europe (Kovats et al., 2014). Thus, further alteration of these attributes will feed back through biological responses to, eventually, alter diversity.

Another approach to understanding global change impacts is to utilize contemporary biological data with forecasted environmental projections to predict future consequences to organisms (e.g., Mair et al., 2018; Mucha et al., 2018; Park et al., 2019). Although such studies are highly illustrative of potential impacts, they are not able to incorporate the actual conditions leading to biological responses caused by global change. This is what sets our results here apart; we can confirm the influence of climate on contemporary changes in fungal diversity, and highlight that both climate means and seasonality‐based shifts will likely impact further richness. Mean temperature increases will likely correspond to increased fungal richness (Fig. 3), assuming availability of appropriate land types (Fig. 2), but only to a threshold after which diversity can be expected to decrease. A decline in tree productivity and precipitation seasonality with future climate change is expected to result in a corresponding decrease in fungal richness.

### Conclusions

Our continuing support of a mid‐latitudinal fungal richness increase is best explained by combining knowledge of land‐use change impacts, tree species diversity, and prevailing climatic and other environmental attributes. The consequences to fungal diversity that the combination of a changing climate and urbanization will have are unequivocal, and collections data are key sources to this understanding.

## AUTHOR CONTRIBUTIONS

C.A. conducted the majority of data preparation, analyses, and writing. All coauthors contributed to data, helped formulate concepts, and edited the manuscript.

## DATA ACCESSIBILITY

Data are accessible through the following URLs: Austria: http://www.univie.ac.at/oemykges/pilzdatenbank/; Switzerland: http://www.swissfungi.ch/; Germany: http://www.pilze-deutschland.de/; Denmark: http://svampe.databasen.org/; Estonia: http://kogud.emu.ee/?do=coll&id=9&lang=eng; Netherlands: https://www.verspreidingsatlas.nl/paddenstoelen; Norway: https://www.gbif.no/datasets/, http://artskart.artsdatabanken.no/Default.aspx; Slovenia: http://www.zdravgozd.si/mikoteka_index_en.aspx; United Kingdom: http://www.fieldmycology.net/, http://www.frdbi.info/

## Supporting information


**APPENDIX S1.** Tukey's honest significant difference (HSD) for multiple comparisons in the types of dynamic land‐cover (ISAM‐HYDE), and whether there is a significant difference in saprotrophic fungal diversity. The significant differences are shaded by values less than 0.05 (orange) or 0.01 (red).

**APPENDIX S2.** Tukey's honest significant difference (HSD) for multiple comparisons in the types of static land‐cover (CLC3), and whether there is a significant difference in saprotrophic fungal diversity. The significant differences are shaded by values less than 0.05 (orange) or 0.01 (red).

**APPENDIX S3.** Tukey's honest significant difference (HSD) for multiple comparisons in the types of dynamic land‐cover (ISAM‐HYDE), and whether there is a significant difference in ectomycorrhizal fungal diversity. The significant differences are shaded by values less than 0.05 (orange) or 0.01 (red).

**APPENDIX S4.** Tukey's honest significant difference (HSD) for multiple comparisons in the types of dynamic land‐cover, and whether there is a significant difference in ectomycorrhizal fungal diversity. The significant differences are shaded by values less than 0.05 (orange) or 0.01 (red).

**APPENDIX S5.** Model specifications, as R script, used for model selection, for both forward and backward procedures. See Methods section for further information and details.

**APPENDIX S6.** The full, initial model output during backward selection processing to predict species richness of saprotrophic fungi.

**APPENDIX S7.** The intermediate model output, with one covariate for each environmental group, for backward selection predicting species richness of saprotrophic fungi.

**APPENDIX S8.** Collinearity correlations, here including easting and northing, between the remaining covariates selected for the final consensus regression model, for saprotrophic fungi. See Methods for further details.

**APPENDIX S9.** The full, initial model output for backward selection predicting species richness of ectomycorrhizal fungi.

**APPENDIX S10.** The intermediate model output, with one covariate for each environmental group, for backward selection predicting species richness of ectomycorrhizal fungi.

**APPENDIX S11.** Collinearity correlations, here including easting and northing, between the remaining covariates selected for the final consensus regression model, for ectomycorrhizal fungi. See Methods for further details.

**APPENDIX S12.** The patterns of the environmental covariate gradients of the data (shaded) are visible as used to predict richness (isolines) of saprotrophic fungi in central to northern Europe. All values are scaled. Lower values are lighter, grading to higher values that are darker.

**APPENDIX S13.** The patterns of the environmental covariate gradients of the data (shaded) are visible as used to predict richness (isolines) of ectomycorrhizal fungi in central to northern Europe. All values are scaled. Lower values are lighter, grading to higher values that are darker.

**APPENDIX S14.** The mean and range in each of the explanatory variables connected to the fruiting records, for the final consensus model for saprotrophic fungi, between each of the land‐use types of the dynamic (ISAM‐HYDE) variable. All variables are scaled.

**APPENDIX S15.** The mean and range in each of the explanatory variables connected to the fruiting records, for the final consensus model for ectomycorrhizal fungi, between each of the land‐use types of the dynamic (ISAM‐HYDE) variable. All variables are scaled.

## APPENDIX 1 The number of species and total collections records for each fungal nutritional mode. Only saprotrophic and ectomycorrhizal fungal designations were investigated further.

**Table:**

Nutritional mode^a^	No. of species	No. of total records
Ectomycorrhizal	1403	1,633,830
Saprotrophic	2593	2,959,513
Mutualistic	20	2928
Mycoparasitic	54	27,990
Parasitic	73	150,478
Unknown	221	80,751

a Mutualistic includes most symbioses of that interaction type, except for ectomycorrhizal (which have their own designation). Parasitic is actually pathogenic or parasitic. Unknown refers to those taxa with unknown mode as well as to those that are possibly known but uncertain. The nutritional modes classification was updated from expert annotations to include more species via FUNGuild designations (accessed 14 March 2018). Species classifications were based off a 2015 version of Index Fungorum (http://www.indexfungorum.org/).

## APPENDIX 3 Number of grids and species totals for the varying amounts of minimal records requirements per grid, for ectomycorrhizal and saprotrophic taxa, as conducted during data preparation procedures. These values can be compared to the 460 total grids across all taxa, which each contained at least 500 records and a total of 4364 species.

**Table:**

Ectomycorrhizal			Saprotrophic		
Grid records minimum	No. of grids	No. of species per grid	Grid records minimum	No. of grids	No. of species per grid
100	449	57–740	—	—	—
200	408	62–740	200	460	121–1307
250	387	62–740	250	459	121–1307
350	356	106–740	350	439	129–1307
500	324	106–740	500	400	129–1307

## APPENDIX 9 ANOVA results from regressions demonstrating the importance of land type on saprotrophic and ectomycorrhizal diversity. Using two sources, one temporally dynamic (ISAM‐HYDE) and one generalized and static (CLC [level 3]), each demonstrated similar levels of significance.

**Table:**

Source	*df*	Sum of squares	Mean squares	*F*	Pr (>*F*)
Saprotrophic richness					
Land cover (ISAM‐HYDE)	8	129,982	16,247.7	15.877	<2.2E‐16
Residuals	365	373,509	1023.3		
Land cover (CLC 3)	11	89,682	8152.9	7.305	2.698E‐11
Residuals	361	402,888	1116.0		
Ectomycorrhizal richness					
Land cover (ISAM‐HYDE)	7	181,685	25,955.0	23.634	<2.2E‐16
Residuals	301	330,556	1098.2		
Land cover (CLC 3)	10	98,273	9827.3	7.184	3.666E‐10
Residuals	299	409,000	1367.9		

Note

*df* = degrees of freedom; Pr (>*F*) = probability of a value greater than *F*.

## APPENDIX 10

Land‐use type impacts on richness for ectomycorrhizal (Ec) and saprotrophic (Sa) fungi, with scaled values for direct comparisons, according to the temporally static land‐type covariate, CLC 3. Values were calculated by classifying each 50 × 50‐km grid based on the highest amount of land type as associated with the fungal collections data by spatial points (single time unit).
