# Supplementary material for: Open‐source data reveal how collections‐based fungal diversity is sensitive to global change
Source: Appl Plant Sci. 2019 Mar 12;7(3):e01227. doi: 10.1002/aps3.1227 (PMC6426159; doi:10.1002/aps3.1227)
Supplement: Supplementary file 1 — APPENDIX S1. Tukey's honest significant difference (HSD) for multiple comparisons in the types of dynamic land‐cover (ISAM‐HYDE), and whether there is a significant difference in saprotrophic fungal diversity. The significant differences are shaded by values less than 0.05 (orange) or 0.01 (red). [file APS3-7-e01227-s001.pdf]

APPENDIX S1. Tukey's honest significant difference (HSD) for multiple comparisons in the types of dynamic land-cover (ISAM-HYDE), and whether there is a significant difference in saprotrophic fungal diversity. The significant differences are shaded by values less than 0.05 (orange) or 0.01 (red).

Comparison		diff	lwr	upr	p.adj
**Cropland**	**Boreal evergr. needleleaf forest**	-30.50	-50.94	-10.07	**0.00**
**Grassland, steppe**	**Boreal evergr. needleleaf forest**	-41.08	-62.07	-20.09	**0.00**
Grassland, steppe	Cropland	-10.58	-25.35	4.19	0.38
**Pastureland**	**Boreal evergr. needleleaf forest**	-51.93	-73.44	-30.43	**0.00**
**Pastureland**	**Cropland**	-21.43	-36.92	-5.95	**0.00**
Pastureland	Grassland, steppe	-10.85	-27.06	5.36	0.48
Temperate decid. broadleaf forest	Boreal evergr. needleleaf forest	26.72	-21.94	75.39	0.74
**Temperate decid. broadleaf forest**	**Cropland**	57.22	10.90	103.55	**0.00**
**Temperate decid. broadleaf forest**	**Grassland, steppe**	67.80	21.23	114.37	**0.00**
**Temperate decid. broadleaf forest**	**Pastureland**	78.66	31.85	125.46	**0.00**
Temperate decid. broadleaf forest	Temperate evergr. needleleaf forest	41.04	-6.07	88.15	0.14
Temperate evergr. needleleaf forest	Boreal evergr. needleleaf forest	-14.32	-36.47	7.83	0.53
Temperate evergr. needleleaf forest	Cropland	16.18	-0.19	32.56	0.06
**Temperate evergr. needleleaf forest**	**Grassland, steppe**	26.76	9.70	43.83	**0.00**
**Temperate evergr. needleleaf forest**	**Pastureland**	37.62	19.93	55.31	**0.00**
Tundra	Boreal evergr. needleleaf forest	-4.67	-78.43	69.09	1.00
Tundra	Cropland	25.83	-46.41	98.07	0.97
Tundra	Grassland, steppe	36.41	-35.99	108.81	0.82
Tundra	Pastureland	47.26	-25.28	119.81	0.52
Tundra	Temperate decid. broadleaf forest	-31.39	-116.06	53.28	0.96
Tundra	Temperate evergr. needleleaf forest	9.65	-63.10	82.39	1.00
Urbanland	Boreal evergr. needleleaf forest	-47.96	-109.06	13.15	0.26
Urbanland	Cropland	-17.45	-76.71	41.80	0.99
Urbanland	Grassland, steppe	-6.87	-66.32	52.58	1.00
Urbanland	Pastureland	3.98	-55.65	63.61	1.00
**Urbanland**	**Temperate decid. broadleaf forest**	-74.68	-148.58	-0.77	**0.05**
Urbanland	Temperate evergr. needleleaf forest	-33.64	-93.51	26.23	0.71
Urbanland	Tundra	-43.28	-135.67	49.10	0.87
**Wetlands, bogs**	**Boreal evergr. needleleaf forest**	-50.09	-88.27	-11.91	**0.00**
Wetlands, bogs	Cropland	-19.59	-54.74	15.56	0.72
Wetlands, bogs	Grassland, steppe	-9.01	-44.48	26.47	1.00
Wetlands, bogs	Pastureland	1.84	-33.93	37.62	1.00
**Wetlands, bogs**	**Temperate decid. broadleaf forest**	-76.81	-133.26	-20.37	**0.00**
Wetlands, bogs	Temperate evergr. needleleaf forest	-35.77	-71.95	0.40	0.06
Wetlands, bogs	Tundra	-45.42	-124.53	33.69	0.69
Wetlands, bogs	Urbanland	-2.13	-69.60	65.33	1.00
