# Supplementary material for: Open‐source data reveal how collections‐based fungal diversity is sensitive to global change
Source: Appl Plant Sci. 2019 Mar 12;7(3):e01227. doi: 10.1002/aps3.1227 (PMC6426159; doi:10.1002/aps3.1227)
Supplement: Supplementary file 2 — APPENDIX S2. Tukey's honest significant difference (HSD) for multiple comparisons in the types of static land‐cover (CLC3), and whether there is a significant difference in saprotrophic fungal diversity. The significant differences are shaded by values less than 0.05 (orange) or 0.01 (red). [file APS3-7-e01227-s002.pdf]

APPENDIX S2. Tukey's honest significant difference (HSD) for multiple comparisons in the types of static land-cover (CLC3), and whether there is a significant difference in saprotrophic fungal diversity. The significant differences are shaded by values less than 0.05 (orange) or 0.01 (red).

Comparison		diff	lwr	upr	p.adj
Arable land (non-irrig.)	Ag. w/natural lands	5.95	-40.59	52.49	1.00
Broadleaved forest	Ag. w/natural lands	29.05	-18.33	76.44	0.68
**Broadleaved forest**	**Arable land (non-irrig.)**	**23.10**	**3.48**	**42.73**	**0.01**
Coniferous forest	Ag. w/natural lands	23.19	-22.88	69.26	0.89
**Coniferous forest**	**Arable land (non-irrig.)**	**17.24**	**1.03**	**33.45**	**0.03**
Coniferous forest	Broadleaved forest	-5.86	-24.36	12.63	1.00
Cultivated patterns	Ag. w/natural lands	15.10	-42.83	73.02	1.00
Cultivated patterns	Arable land (non-irrig.)	9.15	-29.52	47.81	1.00
Cultivated patterns	Broadleaved forest	-13.96	-53.63	25.72	0.99
Cultivated patterns	Coniferous forest	-8.09	-46.20	30.01	1.00
Mixed forest	Ag. w/natural lands	28.25	-19.71	76.22	0.73
**Mixed forest**	**Arable land (non-irrig.)**	**22.30**	**1.32**	**43.29**	**0.03**
Mixed forest	Broadleaved forest	-0.80	-23.60	22.00	1.00
Mixed forest	Coniferous forest	5.06	-14.87	25.00	1.00
Mixed forest	Cultivated patterns	13.16	-27.21	53.52	1.00
Moors and heathland	Ag. w/natural lands	-13.61	-70.37	43.14	1.00
Moors and heathland	Arable land (non-irrig.)	-19.56	-56.45	17.32	0.85
**Moors and heathland**	**Broadleaved forest**	**-42.67**	**-80.61**	**-4.72**	**0.01**
**Moors and heathland**	**Coniferous forest**	**-36.80**	**-73.10**	**-0.50**	**0.04**
Moors and heathland	Cultivated patterns	-28.71	-79.20	21.79	0.78
**Moors and heathland**	**Mixed forest**	**-41.87**	**-80.54**	**-3.20**	**0.02**
Natural grasslands	Ag. w/natural lands	-21.39	-84.84	42.06	0.99
Natural grasslands	Arable land (non-irrig.)	-27.34	-73.88	19.20	0.74
**Natural grasslands**	**Broadleaved forest**	**-50.45**	**-97.83**	**-3.06**	**0.03**
Natural grasslands	Coniferous forest	-44.58	-90.66	1.49	0.07
Natural grasslands	Cultivated patterns	-36.49	-94.41	21.43	0.64
**Natural grasslands**	**Mixed forest**	**-49.65**	**-97.61**	**-1.68**	**0.04**
Natural grasslands	Moors and heathland	-7.78	-64.53	48.97	1.00
Pastures	Ag. w/natural lands	-14.08	-62.19	34.03	1.00
Pastures	Arable land (non-irrig.)	-20.03	-41.36	1.30	0.09
**Pastures**	**Broadleaved forest**	**-43.13**	**-66.25**	**-20.02**	**0.00**
**Pastures**	**Coniferous forest**	**-37.27**	**-57.56**	**-16.98**	**0.00**
Pastures	Cultivated patterns	-29.18	-69.72	11.37	0.43
**Pastures**	**Mixed forest**	**-42.33**	**-66.61**	**-18.05**	**0.00**
Pastures	Moors and heathland	-0.47	-39.32	38.39	1.00
Pastures	Natural grasslands	7.31	-40.80	55.43	1.00
Peat bogs	Ag. w/natural lands	-40.56	-130.29	49.17	0.94
Peat bogs	Arable land (non-irrig.)	-46.51	-125.20	32.18	0.73
Peat bogs	Broadleaved forest	-69.61	-148.80	9.58	0.15
Peat bogs	Coniferous forest	-63.75	-142.16	14.67	0.24
Peat bogs	Cultivated patterns	-55.65	-141.57	30.26	0.60
Peat bogs	Mixed forest	-68.81	-148.35	10.73	0.17
Peat bogs	Moors and heathland	-26.95	-112.07	58.18	1.00
Peat bogs	Natural grasslands	-19.17	-108.90	70.57	1.00
Peat bogs	Pastures	-26.48	-106.11	53.15	0.99
Transition wood/shrubland	Ag. w/natural lands	28.70	-37.84	95.25	0.96
Transition wood/shrubland	Arable land (non-irrig.)	22.75	-27.93	73.43	0.95
Transition wood/shrubland	Broadleaved forest	-0.35	-51.81	51.11	1.00
Transition wood/shrubland	Coniferous forest	5.51	-44.74	55.77	1.00
Transition wood/shrubland	Cultivated patterns	13.61	-47.69	74.91	1.00
Transition wood/shrubland	Mixed forest	0.45	-51.54	52.44	1.00
Transition wood/shrubland	Moors and heathland	42.32	-17.88	102.51	0.47
Transition wood/shrubland	Natural grasslands	50.10	-16.45	116.64	0.36
Transition wood/shrubland	Pastures	42.78	-9.35	94.91	0.23
Transition wood/shrubland	Peat bogs	69.26	-22.69	161.21	0.36
Urban fabric	Ag. w/natural lands	11.82	-43.13	66.77	1.00
Urban fabric	Arable land (non-irrig.)	5.87	-28.18	39.92	1.00
Urban fabric	Broadleaved forest	-17.23	-52.43	17.96	0.90
Urban fabric	Coniferous forest	-11.37	-44.78	22.04	0.99
Urban fabric	Cultivated patterns	-3.28	-51.74	45.19	1.00
Urban fabric	Mixed forest	-16.43	-52.41	19.54	0.94
Urban fabric	Moors and heathland	25.43	-21.62	72.49	0.83
Urban fabric	Natural grasslands	33.21	-21.74	88.16	0.70
Urban fabric	Pastures	25.90	-10.27	62.07	0.44
Urban fabric	Peat bogs	52.38	-31.56	136.32	0.66
Urban fabric	Transition wood/shrubland	-16.88	-75.38	41.62	1.00
