# Supplementary material for: Open‐source data reveal how collections‐based fungal diversity is sensitive to global change
Source: Appl Plant Sci. 2019 Mar 12;7(3):e01227. doi: 10.1002/aps3.1227 (PMC6426159; doi:10.1002/aps3.1227)
Supplement: Supplementary file 3 — APPENDIX S3. Tukey's honest significant difference (HSD) for multiple comparisons in the types of dynamic land‐cover (ISAM‐HYDE), and whether there is a significant difference in ectomycorrhizal fungal diversity. The significant differences are shaded by values less than 0.05 (orange) or 0.01 (red). [file APS3-7-e01227-s003.pdf]

APPENDIX S3. Tukey's honest significant difference (HSD) for multiple comparisons in the types of dynamic land-cover (ISAM-HYDE), and whether there is a significant difference in ectomycorrhizal fungal diversity. The significant differences are shaded by values less than 0.05 (orange) or 0.01 (red).

Comparison		diff	lwr	upr	p.adj
**Cropland**	**Boreal evergr. needleleaf forest**	-46.11	-70.68	-21.53	0.00
**Grassland, steppe**	**Boreal evergr. needleleaf forest**	-54.44	-80.37	-28.50	0.00
Grassland, steppe	Cropland	-8.33	-25.77	9.10	0.86
**Pastureland**	**Boreal evergr. needleleaf forest**	-62.06	-88.05	-36.06	0.00
Pastureland	Cropland	-15.95	-33.48	1.58	0.11
Pastureland	Grassland, steppe	-7.62	-27.01	11.77	0.95
Temperate decid. broadleaf forest	Boreal evergr. needleleaf forest	-8.39	-34.45	17.66	0.99
**Temperate decid. broadleaf forest**	**Cropland**	37.71	20.09	55.33	0.00
**Temperate decid. broadleaf forest**	**Grassland, steppe**	46.04	26.57	65.51	0.00
**Temperate decid. broadleaf forest**	**Pastureland**	53.66	34.11	73.22	0.00
Temperate evergr. needleleaf forest	Boreal evergr. needleleaf forest	7.70	-44.48	59.88	1.00
**Temperate evergr. needleleaf forest**	**Cropland**	53.80	5.29	102.32	0.02
**Temperate evergr. needleleaf forest**	**Grassland, steppe**	62.14	12.92	111.36	0.00
**Temperate evergr. needleleaf forest**	**Pastureland**	69.76	20.50	119.01	0.00
Temperate evergr. needleleaf forest	Temperate decid. broadleaf forest	16.09	-33.19	65.38	0.98
Tundra	Boreal evergr. needleleaf forest	26.47	-81.55	134.48	1.00
Tundra	Cropland	72.57	-33.73	178.87	0.45
Tundra	Grassland, steppe	80.90	-25.72	187.53	0.30
Tundra	Pastureland	88.52	-18.11	195.16	0.19
Tundra	Temperate decid. broadleaf forest	34.86	-71.79	141.51	0.98
Tundra	Temperate evergr. needleleaf forest	18.77	-97.07	134.60	1.00
**Urbanland**	**Boreal evergr. needleleaf forest**	-80.81	-145.72	-15.90	0.00
Urbanland	Cropland	-34.70	-96.71	27.30	0.72
Urbanland	Grassland, steppe	-26.37	-88.93	36.19	0.93
Urbanland	Pastureland	-18.75	-81.34	43.83	0.99
**Urbanland**	**Temperate decid. broadleaf forest**	-72.41	-135.02	-9.80	0.01
**Urbanland**	**Temperate evergr. needleleaf forest**	-88.51	-165.73	-11.28	0.01
Urbanland	Tundra	-107.28	-229.38	14.83	0.14
**Wetlands and bogs**	**Boreal evergr. needleleaf forest**	-59.40	-107.87	-10.93	0.00
Wetlands and bogs	Cropland	-13.29	-57.81	31.22	0.99
Wetlands and bogs	Grassland, steppe	-4.96	-50.24	40.31	1.00
Wetlands and bogs	Pastureland	2.66	-42.65	47.97	1.00
**Wetlands and bogs**	**Temperate decid. broadleaf forest**	-51.01	-96.35	-5.66	0.01
**Wetlands and bogs**	**Temperate evergr. needleleaf forest**	-67.10	-131.13	-3.07	0.03
Wetlands and bogs	Tundra	-85.87	-200.08	28.35	0.32
Wetlands and bogs	Urbanland	21.41	-53.36	96.18	0.99
