# Supplementary material for: Open‐source data reveal how collections‐based fungal diversity is sensitive to global change
Source: Appl Plant Sci. 2019 Mar 12;7(3):e01227. doi: 10.1002/aps3.1227 (PMC6426159; doi:10.1002/aps3.1227)
Supplement: Supplementary file 4 — APPENDIX S4. Tukey's honest significant difference (HSD) for multiple comparisons in the types of dynamic land‐cover, and whether there is a significant difference in ectomycorrhizal fungal diversity. The significant differences are shaded by values less than 0.05 (orange) or 0.01 (red). [file APS3-7-e01227-s004.pdf]

APPENDIX S4. Tukey's honest significant difference (HSD) for multiple comparisons in the types of dynamic land-cover, and whether there is a significant difference in ectomycorrhizal fungal diversity. The significant differences are shaded by values less than 0.05 (orange) or 0.01 (red).

Comparison		diff	lwr	upr	p.adj
Arable land (non-irrig.)	Ag. w/natural lands	0.40	-50.73	51.52	1.00
Broadleaved forest	Ag. w/natural lands	25.63	-26.45	77.70	0.88
**Broadleaved forest**	**Arable land (non-irrig.)**	25.23	2.25	48.20	**0.02**
Coniferous forest	Ag. w/natural lands	29.76	-20.67	80.19	0.71
**Coniferous forest**	**Arable land (non-irrig.)**	29.36	10.42	48.30	**0.00**
Coniferous forest	Broadleaved forest	4.14	-17.24	25.51	1.00
Cultivated patterns	Ag. w/natural lands	3.94	-62.81	70.69	1.00
Cultivated patterns	Arable land (non-irrig.)	3.54	-44.11	51.20	1.00
Cultivated patterns	Broadleaved forest	-21.69	-70.36	26.99	0.94
Cultivated patterns	Coniferous forest	-25.82	-72.73	21.09	0.79
Mixed forest	Ag. w/natural lands	36.64	-16.16	89.44	0.47
**Mixed forest**	**Arable land (non-irrig.)**	36.24	11.66	60.81	**0.00**
Mixed forest	Broadleaved forest	11.01	-15.48	37.50	0.96
Mixed forest	Coniferous forest	6.87	-16.21	29.96	1.00
Mixed forest	Cultivated patterns	32.70	-16.75	82.15	0.55
Moors and heathland	Ag. w/natural lands	9.31	-63.34	81.96	1.00
Moors and heathland	Arable land (non-irrig.)	8.91	-46.71	64.53	1.00
Moors and heathland	Broadleaved forest	-16.32	-72.81	40.18	1.00
Moors and heathland	Coniferous forest	-20.45	-75.43	34.52	0.98
Moors and heathland	Cultivated patterns	5.37	-64.88	75.62	1.00
Moors and heathland	Mixed forest	-27.33	-84.49	29.84	0.90
Natural grasslands	Ag. w/natural lands	-43.36	-128.20	41.47	0.86
Natural grasslands	Arable land (non-irrig.)	-43.76	-114.56	27.04	0.65
Natural grasslands	Broadleaved forest	-68.99	-140.48	2.50	0.07
**Natural grasslands**	**Coniferous forest**	-73.13	-143.42	-2.83	**0.03**
Natural grasslands	Cultivated patterns	-47.30	-130.10	35.49	0.75
**Natural grasslands**	**Mixed forest**	-80.00	-152.02	-7.98	**0.02**
Natural grasslands	Moors and heathland	-52.67	-140.29	34.95	0.68

Comparison		diff	lwr	upr	p.adj
Pastures	Ag. w/natural lands	-15.12	-69.66	39.42	1.00
Pastures	Arable land (non-irrig.)	-15.52	-43.63	12.60	0.79
**Pastures**	**Broadleaved forest**	-40.74	-70.55	-10.93	**0.00**
**Pastures**	**Coniferous forest**	-44.88	-71.71	-18.05	**0.00**
Pastures	Cultivated patterns	-19.06	-70.36	32.24	0.98
**Pastures**	**Mixed forest**	-51.75	-82.82	-20.69	**0.00**
Pastures	Moors and heathland	-24.43	-83.20	34.35	0.96
Pastures	Natural grasslands	28.25	-45.06	101.55	0.98
Transition wood/shrubland	Ag. w/natural lands	42.57	-34.88	120.01	0.79
Transition wood/shrubland	Arable land (non-irrig.)	42.17	-19.58	103.92	0.50
Transition wood/shrubland	Broadleaved forest	16.94	-45.60	79.48	1.00
Transition wood/shrubland	Coniferous forest	12.81	-48.37	73.98	1.00
Transition wood/shrubland	Cultivated patterns	38.63	-36.57	113.82	0.85
Transition wood/shrubland	Mixed forest	5.93	-57.22	69.08	1.00
Transition wood/shrubland	Moors and heathland	33.26	-47.22	113.74	0.96
Transition wood/shrubland	Natural grasslands	85.93	-5.70	177.56	0.09
Transition wood/shrubland	Pastures	57.69	-6.92	122.29	0.13
Urban fabric	Ag. w/natural lands	-1.09	-63.04	60.86	1.00
Urban fabric	Arable land (non-irrig.)	-1.49	-42.16	39.18	1.00
Urban fabric	Broadleaved forest	-26.72	-68.58	15.14	0.60
Urban fabric	Coniferous forest	-30.85	-70.64	8.94	0.30
Urban fabric	Cultivated patterns	-5.03	-64.15	54.09	1.00
Urban fabric	Mixed forest	-37.73	-80.49	5.03	0.14
Urban fabric	Moors and heathland	-10.40	-76.11	55.32	1.00
Urban fabric	Natural grasslands	42.27	-36.70	121.25	0.82
Urban fabric	Pastures	14.03	-30.86	58.92	1.00
Urban fabric	Transition wood/shrubland	-43.66	-114.63	27.32	0.65
