# Supplementary material for: Open‐source data reveal how collections‐based fungal diversity is sensitive to global change
Source: Appl Plant Sci. 2019 Mar 12;7(3):e01227. doi: 10.1002/aps3.1227 (PMC6426159; doi:10.1002/aps3.1227)
Supplement: Supplementary file 5 — APPENDIX S5. Model specifications, as R script, used for model selection, for both forward and backward procedures. See Methods section for further information and details. [file APS3-7-e01227-s005.docx]

**APPENDIX S5.** Model specifications, as R script, used for model selection, for both forward and backward procedures. See Methods section for further information and details.

MODEL <- gamm(data = DATA, Scaled_Richness ~

s(Scaled_easting, Scaled_northing, k = 15, bs = "tp") +

s(Variables, k = 10, bs = "tp") + s(Variables, k = 10, bs = "tp") + …,

offset=(log(RecordsPerGrid)),

weights = varPower(form =~ 1/RecordsPerGrid),

random = list(LandCover = ~1), family="gaussian",

correlation=corExp(form =~ Scaled_easting + Scaled_northing, nugget = TRUE))

## internal model diagnostics:

AIC(MODEL$lme)

plot(Variogram(MODEL$lme,

form =~(Scaled_easting + Scaled_northing) | LandCoverAnnual,

data = DATA, nugget = TRUE, maxDist = 200000, resType = "normalized"))

TEMP_MODEL <- as.data.frame(cbind(

resid(MODEL$lme), DATA$Scaled_easting, DATA$Scaled_northing), stringsAsFactors = FALSE)

colnames(TEMP_MODEL) <- c("Residuals", "Scaled_easting", "Scaled_northing")

coordinates(TEMP_MODEL) <- c("Scaled_easting", "Scaled_northing")

bubble(TEMP_MODEL, "Residuals", col=c("black", "grey"), main="Normalized residuals", xlab="Easting (UTM scaled)",ylab="Northing (UTM scaled)")

rm(TEMP_MODEL)
