# Supplementary material for: Open‐source data reveal how collections‐based fungal diversity is sensitive to global change
Source: Appl Plant Sci. 2019 Mar 12;7(3):e01227. doi: 10.1002/aps3.1227 (PMC6426159; doi:10.1002/aps3.1227)
Supplement: Supplementary file 6 — APPENDIX S6. The full, initial model output during backward selection processing to predict species richness of saprotrophic fungi. [file APS3-7-e01227-s006.docx]

**APPENDIX S6.** The full, initial model output during backward selection processing to predict species richness of saprotrophic fungi.

Family: gaussian

Link function: identity

Formula:

Scaled_SaproRichness ~ s(Scaled_UTM_easting, Scaled_UTM_northing, k = 15, bs = "tp") + s(Scaled_Altitude, k = 10, bs = "tp") + s(Scaled_TreeSpp_All, k = 10, bs = "tp") + s(Scaled_PrecipSeasonality_bio15, k = 10, bs = "tp") + s(Scaled_PrecipWettestQrtr_bio16, k = 10, bs = "tp") + s(Scaled_PrecipCollectionDay, k = 10, bs = "tp") +

s(Scaled_MeanTemp_bio1, k = 10, bs = "tp") + s(Scaled_TempWarmestQrtr_bio10, k = 10, bs = "tp") + s(Scaled_TempCollectionDay, k = 10, bs = "tp") + s(Scaled_TempWettestQrtr_bio8, k = 10, bs = "tp") +

s(Scaled_DiurnalTempRange_bio2, k = 10, bs = "tp") +

s(Scaled_Isothermality_bio3, k = 10, bs = "tp") + s(Scaled_AnnualTempRange_bio7, k = 10, bs = "tp") + s(Scaled_NDVI_MeanAnnual, k = 10, bs = "tp") +

s(Scaled_NHx_AnnualMax, k = 10, bs = "tp") + s(Scaled_NOy_AnnualMax, k = 10, bs = "tp") + s(Scaled_SoilOrgCarbon, k = 10, bs = "tp")

Parametric coefficients:

Estimate Std. Error t value Pr(>|t|)

(Intercept) 0.01797 0.05154 0.349 0.728

Approximate significance of smooth terms:

edf Ref.df F p-value

s(Scaled_UTM_easting,Scaled_UTM_northing) 7.261 7.261 3.575 0.000863 ***

s(Scaled_Altitude) 1.000 1.000 0.063 0.801786

s(Scaled_TreeSpp_All) 1.000 1.000 0.497 0.481384

s(Scaled_PrecipSeasonality_bio15) 2.050 2.050 2.146 0.124126

s(Scaled_PrecipWettestQrtr_bio16) 1.000 1.000 1.109 0.292991

s(Scaled_PrecipCollectionDay) 1.000 1.000 3.217 0.073702 .

s(Scaled_MeanTemp_bio1) 3.514 3.514 7.289 5.68e-05 ***

s(Scaled_TempWarmestQrtr_bio10) 1.000 1.000 0.633 0.426649

s(Scaled_TempCollectionDay) 1.000 1.000 1.503 0.220994

s(Scaled_TempWettestQrtr_bio8) 1.000 1.000 0.613 0.434354

s(Scaled_DiurnalTempRange_bio2) 1.000 1.000 0.026 0.872188

s(Scaled_Isothermality_bio3) 6.119 6.119 3.555 0.002265 **

s(Scaled_AnnualTempRange_bio7) 1.000 1.000 0.374 0.541201

s(Scaled_NDVI_MeanAnnual) 1.000 1.000 9.478 0.002237 **

s(Scaled_NHx_AnnualMax) 1.000 1.000 1.721 0.190449

s(Scaled_NOy_AnnualMax) 1.000 1.000 2.249 0.134544

s(Scaled_SoilOrgCarbon) 1.000 1.000 2.491 0.115378

---

Signif. codes: 0 '***' 0.001 '**' 0.01 '*' 0.05 '.' 0.1 ' ' 1

R-sq.(adj) = 0.426

Scale est. = 3.8989 n = 389
