# Supplementary material for: Open‐source data reveal how collections‐based fungal diversity is sensitive to global change
Source: Appl Plant Sci. 2019 Mar 12;7(3):e01227. doi: 10.1002/aps3.1227 (PMC6426159; doi:10.1002/aps3.1227)
Supplement: Supplementary file 7 — APPENDIX S7. The intermediate model output, with one covariate for each environmental group, for backward selection predicting species richness of saprotrophic fungi. [file APS3-7-e01227-s007.docx]

**APPENDIX S7.** The intermediate model output, with one covariate for each environmental group, for backward selection predicting species richness of saprotrophic fungi.

Parametric coefficients:

Estimate Std. Error t value Pr(>|t|)

(Intercept) 0.01951 0.05532 0.353 0.724

Approximate significance of smooth terms:

edf Ref.df F p-value

s(Scaled_UTM_easting,Scaled_UTM_northing) 8.967 8.968 3.727 0.000163 ***

s(Scaled_Altitude) 1.671 1.671 0.546 0.409137

s(Scaled_TreeSpp_All) 1.000 1.000 0.797 0.372477

s(Scaled_PrecipSeasonality_bio15) 2.275 2.275 2.695 0.083211 .

s(Scaled_PrecipWettestQrtr_bio16) 1.000 1.000 0.978 0.323299

s(Scaled_PrecipCollectionDay) 1.000 1.000 3.860 0.050192 .

s(Scaled_MeanTemp_bio1) 3.384 3.384 6.808 0.000101 ***

s(Scaled_TempWettestQrtr_bio8) 1.000 1.000 0.387 0.534476

s(Scaled_AnnualTempRange_bio7) 1.000 1.000 13.291 0.000305 ***

s(Scaled_NDVI_MeanAnnual) 1.000 1.000 6.850 0.009224 **

s(Scaled_NOy_AnnualMax) 1.000 1.000 4.681 0.031129 *

s(Scaled_SoilOrgCarbon) 1.000 1.000 1.968 0.161470

---

Signif. codes: 0 '***' 0.001 '**' 0.01 '*' 0.05 '.' 0.1 ' ' 1

R-sq.(adj) = 0.394

Scale est. = 4.1259 n = 389
