# Supplementary material for: Open‐source data reveal how collections‐based fungal diversity is sensitive to global change
Source: Appl Plant Sci. 2019 Mar 12;7(3):e01227. doi: 10.1002/aps3.1227 (PMC6426159; doi:10.1002/aps3.1227)

Andrew et al.—Applications in Plant Sciences 2019 7(3)—Data Supplement S8
DOI

APPENDIX S8. Collinearity correlations, here including easting and northing, between the remaining covariates selected for the final consensus regression model, for saprotrophic fungi. See Methods for further details.

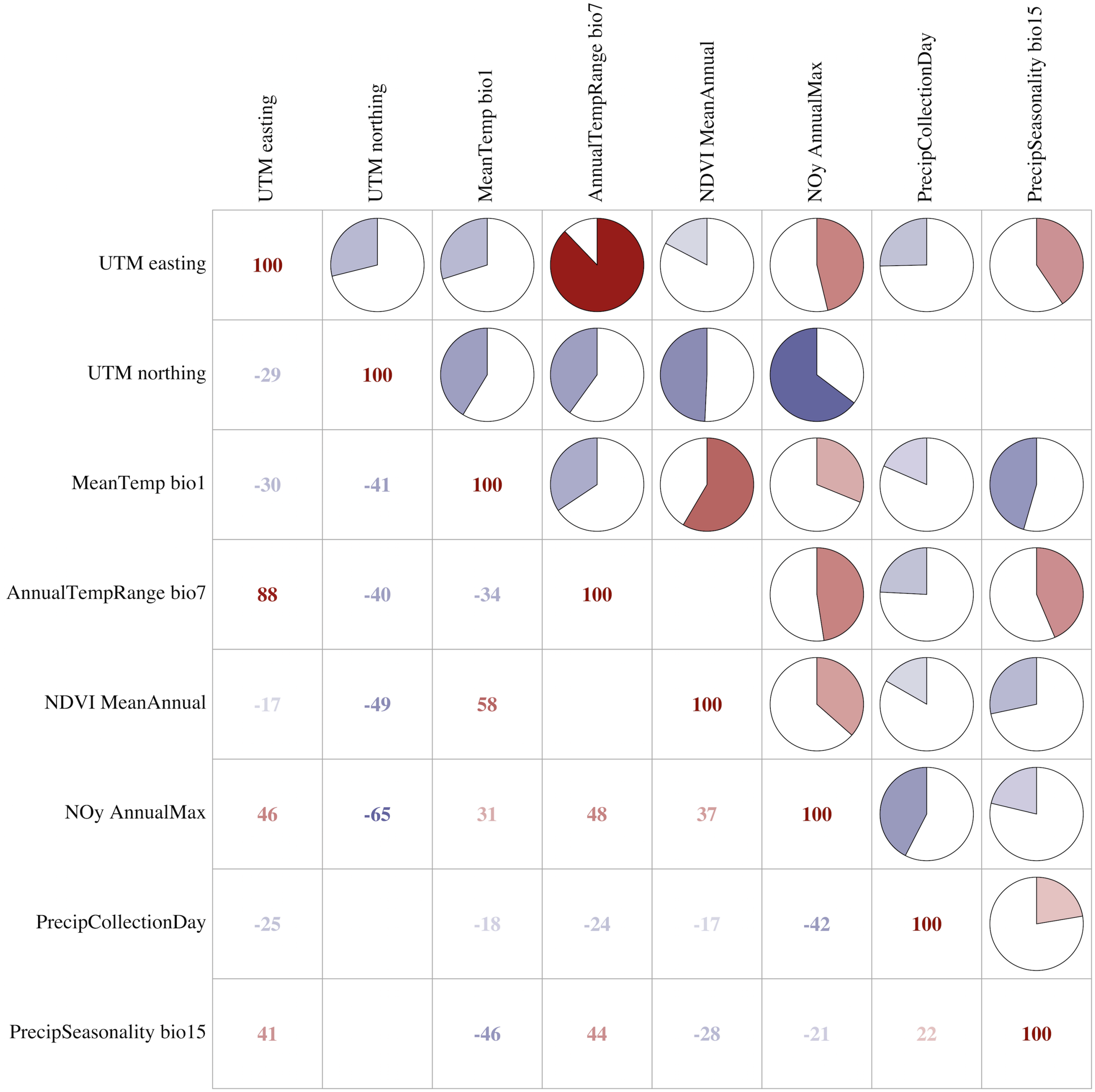

Supplement: Supplementary file 8 — APPENDIX S8. Collinearity correlations, here including easting and northing, between the remaining covariates selected for the final consensus regression model, for saprotrophic fungi. See Methods for further details. [file APS3-7-e01227-s008.pdf]
