# Supplementary material for: Open‐source data reveal how collections‐based fungal diversity is sensitive to global change
Source: Appl Plant Sci. 2019 Mar 12;7(3):e01227. doi: 10.1002/aps3.1227 (PMC6426159; doi:10.1002/aps3.1227)
Supplement: Supplementary file 10 — APPENDIX S10. The intermediate model output, with one covariate for each environmental group, for backward selection predicting species richness of ectomycorrhizal fungi. [file APS3-7-e01227-s010.docx]

**APPENDIX S10.** The intermediate model output, with one covariate for each environmental group, for backward selection predicting species richness of ectomycorrhizal fungi.

Family: gaussian

Link function: identity

Formula:

Scaled_EctoRichness ~ s(Scaled_UTM_easting, Scaled_UTM_northing, k = 15, bs = "tp") + s(Scaled_Altitude, k = 10, bs = "tp") + s(Scaled_TreeSpp_Ecto, k = 10, bs = "tp") + s(Scaled_PrecipSeasonality_bio15, k = 10, bs = "tp") + s(Scaled_PrecipWettestQrtr_bio16, k = 10, bs = "tp") + s(Scaled_PrecipCollectionDay, k = 10, bs = "tp") +

s(Scaled_MeanTemp_bio1, k = 10, bs = "tp") + s(Scaled_TempWarmestQrtr_bio10, k = 10, bs = "tp") + s(Scaled_TempCollectionDay, k = 10, bs = "tp") + s(Scaled_TempWettestQrtr_bio8, k = 10, bs = "tp") +

s(Scaled_DiurnalTempRange_bio2, k = 10, bs = "tp") + s(Scaled_Isothermality_bio3, k = 10, bs = "tp") + s(Scaled_AnnualTempRange_bio7, k = 10, bs = "tp") + s(Scaled_NDVI_MeanAnnual, k = 10, bs = "tp") +

s(Scaled_NHx_AnnualMax, k = 10, bs = "tp") + s(Scaled_NOy_AnnualMax, k = 10, bs = "tp") + s(Scaled_SoilOrgCarbon, k = 10, bs = "tp")

Parametric coefficients:

Estimate Std. Error t value Pr(>|t|)

(Intercept) 0.009017 0.045056 0.2 0.842

Approximate significance of smooth terms:

edf Ref.df F p-value

s(Scaled_UTM_easting,Scaled_UTM_northing) 10.050 10.050 3.425 0.000278 ***

s(Scaled_Altitude) 1.001 1.001 0.005 0.943993

s(Scaled_TreeSpp_Ecto) 1.000 1.000 2.647 0.104796

s(Scaled_PrecipSeasonality_bio15) 2.740 2.740 3.404 0.080910 .

s(Scaled_PrecipWettestQrtr_bio16) 1.000 1.000 0.696 0.404921

s(Scaled_PrecipCollectionDay) 1.000 1.000 0.103 0.748273

s(Scaled_MeanTemp_bio1) 4.403 4.403 7.264 8.91e-06 ***

s(Scaled_TempWarmestQrtr_bio10) 1.000 1.000 1.766 0.184959

s(Scaled_TempCollectionDay) 1.000 1.000 0.082 0.774978

s(Scaled_TempWettestQrtr_bio8) 1.000 1.000 0.402 0.526603

s(Scaled_DiurnalTempRange_bio2) 1.000 1.000 1.322 0.251112

s(Scaled_Isothermality_bio3) 1.000 1.000 2.251 0.134640

s(Scaled_AnnualTempRange_bio7) 1.000 1.000 1.134 0.287726

s(Scaled_NDVI_MeanAnnual) 1.000 1.000 6.161 0.013622 *

s(Scaled_NHx_AnnualMax) 1.000 1.000 0.441 0.507017

s(Scaled_NOy_AnnualMax) 1.000 1.000 1.666 0.197838

s(Scaled_SoilOrgCarbon) 1.000 1.000 3.261 0.071973 .

---

Signif. codes: 0 '***' 0.001 '**' 0.01 '*' 0.05 '.' 0.1 ' ' 1

R-sq.(adj) = 0.63

Scale est. = 2.7665 n = 319
