# Supplementary material for: Open‐source data reveal how collections‐based fungal diversity is sensitive to global change
Source: Appl Plant Sci. 2019 Mar 12;7(3):e01227. doi: 10.1002/aps3.1227 (PMC6426159; doi:10.1002/aps3.1227)

APPENDIX S11. Collinearity correlations, here including easting and northing, between the remaining covariates selected for the final consensus regression model, for ectomycorrhizal fungi. See Methods for further details.

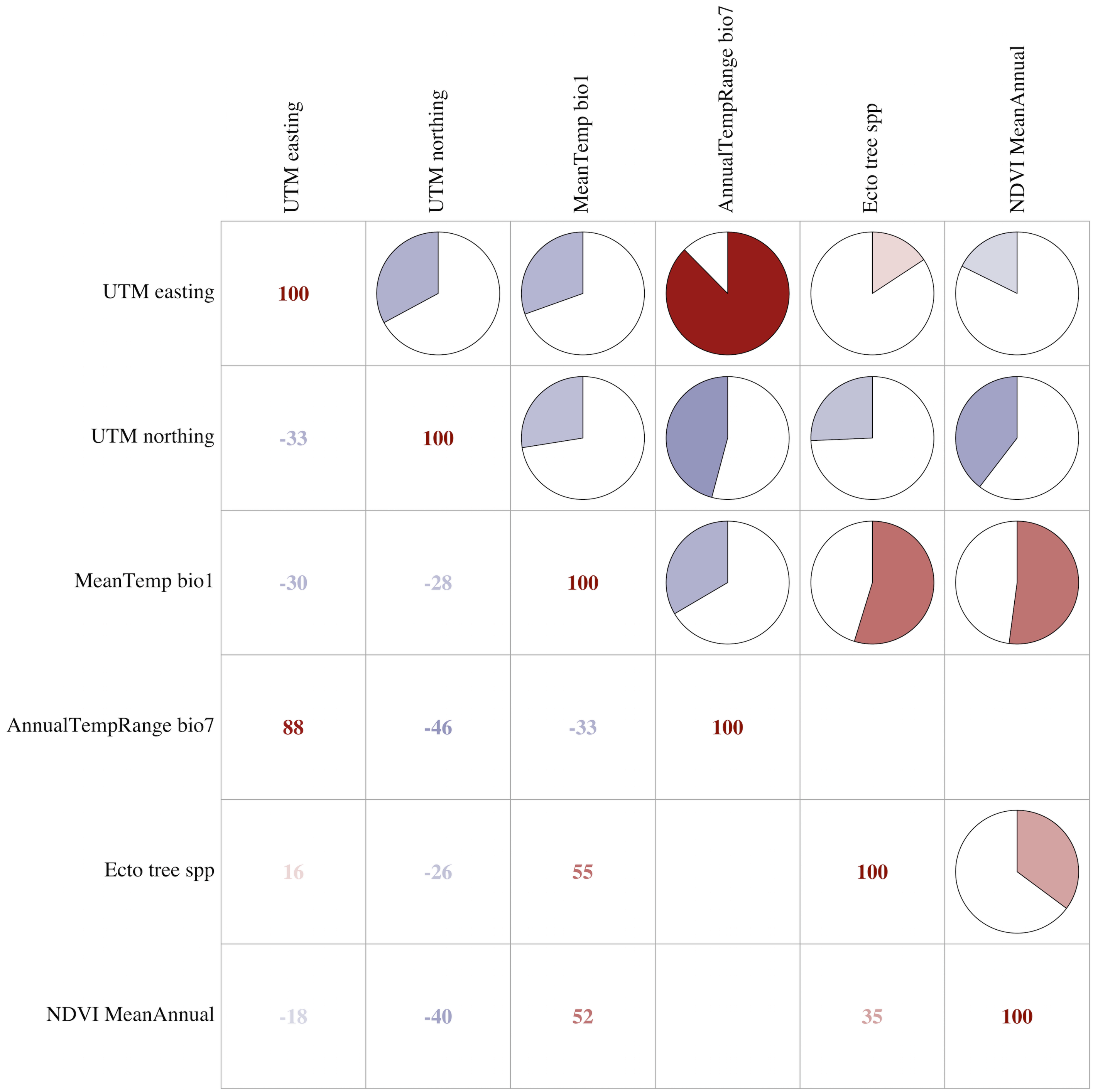

Supplement: Supplementary file 11 — APPENDIX S11. Collinearity correlations, here including easting and northing, between the remaining covariates selected for the final consensus regression model, for ectomycorrhizal fungi. See Methods for further details. [file APS3-7-e01227-s011.pdf]
