# Supplementary material for: Open‐source data reveal how collections‐based fungal diversity is sensitive to global change
Source: Appl Plant Sci. 2019 Mar 12;7(3):e01227. doi: 10.1002/aps3.1227 (PMC6426159; doi:10.1002/aps3.1227)

APPENDIX S13. The patterns of the environmental covariate gradients of the data (shaded) are visible as used to predict richness (isolines) of ectomycorrhizal fungi in central to northern Europe. All values are scaled. Lower values are lighter, grading to higher values that are darker.

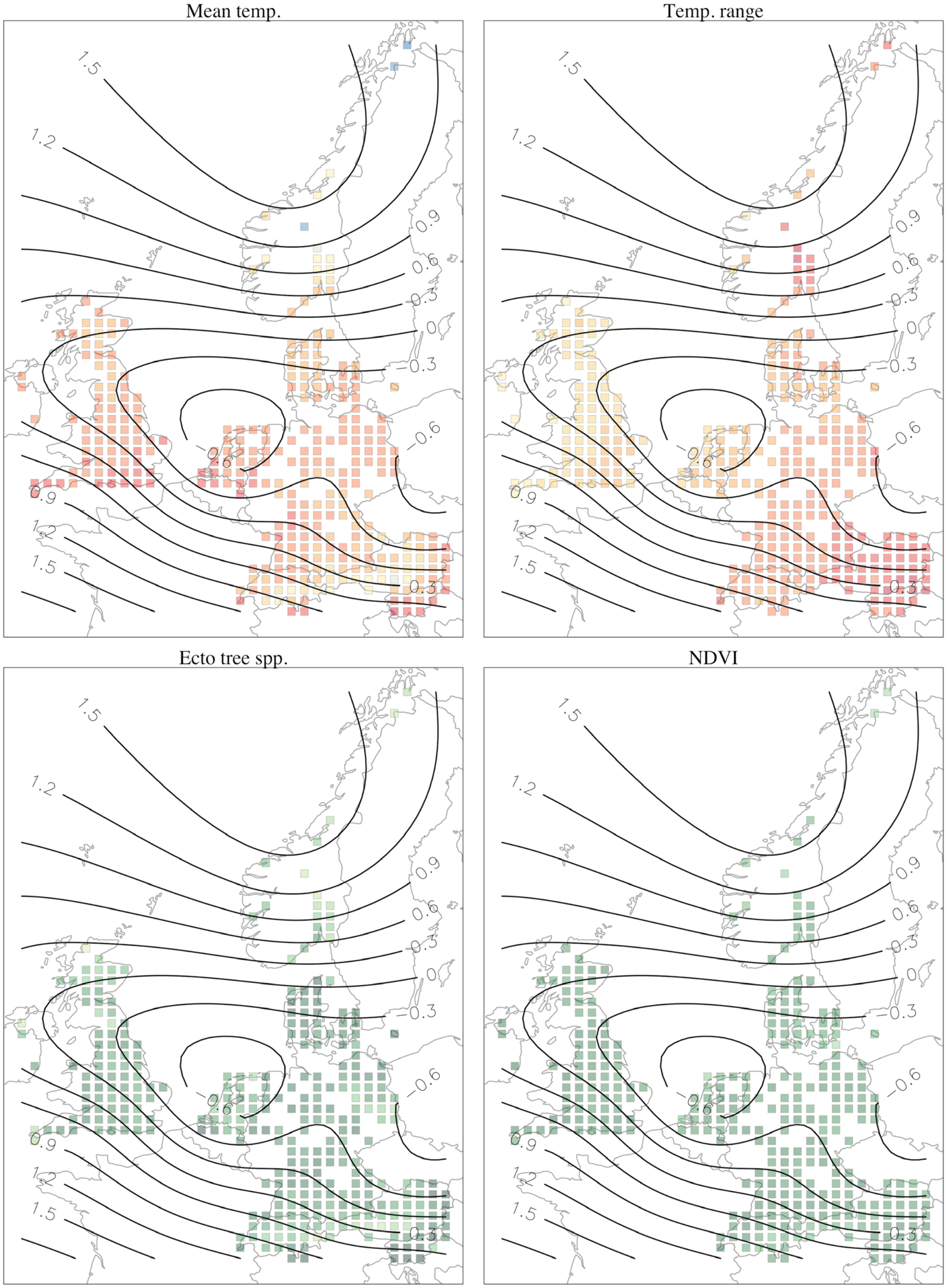

Supplement: Supplementary file 13 — APPENDIX S13. The patterns of the environmental covariate gradients of the data (shaded) are visible as used to predict richness (isolines) of ectomycorrhizal fungi in central to northern Europe. All values are scaled. Lower values are lighter, grading to higher values that are darker. [file APS3-7-e01227-s013.pdf]
