# Supplementary figures and images for: Open‐source data reveal how collections‐based fungal diversity is sensitive to global change
Source: Appl Plant Sci. 2019 Mar 12;7(3):e01227. doi: 10.1002/aps3.1227 (PMC6426159; doi:10.1002/aps3.1227)

land-use types of the dynamic (ISAM-HYDE) variable. All variables are scaled.

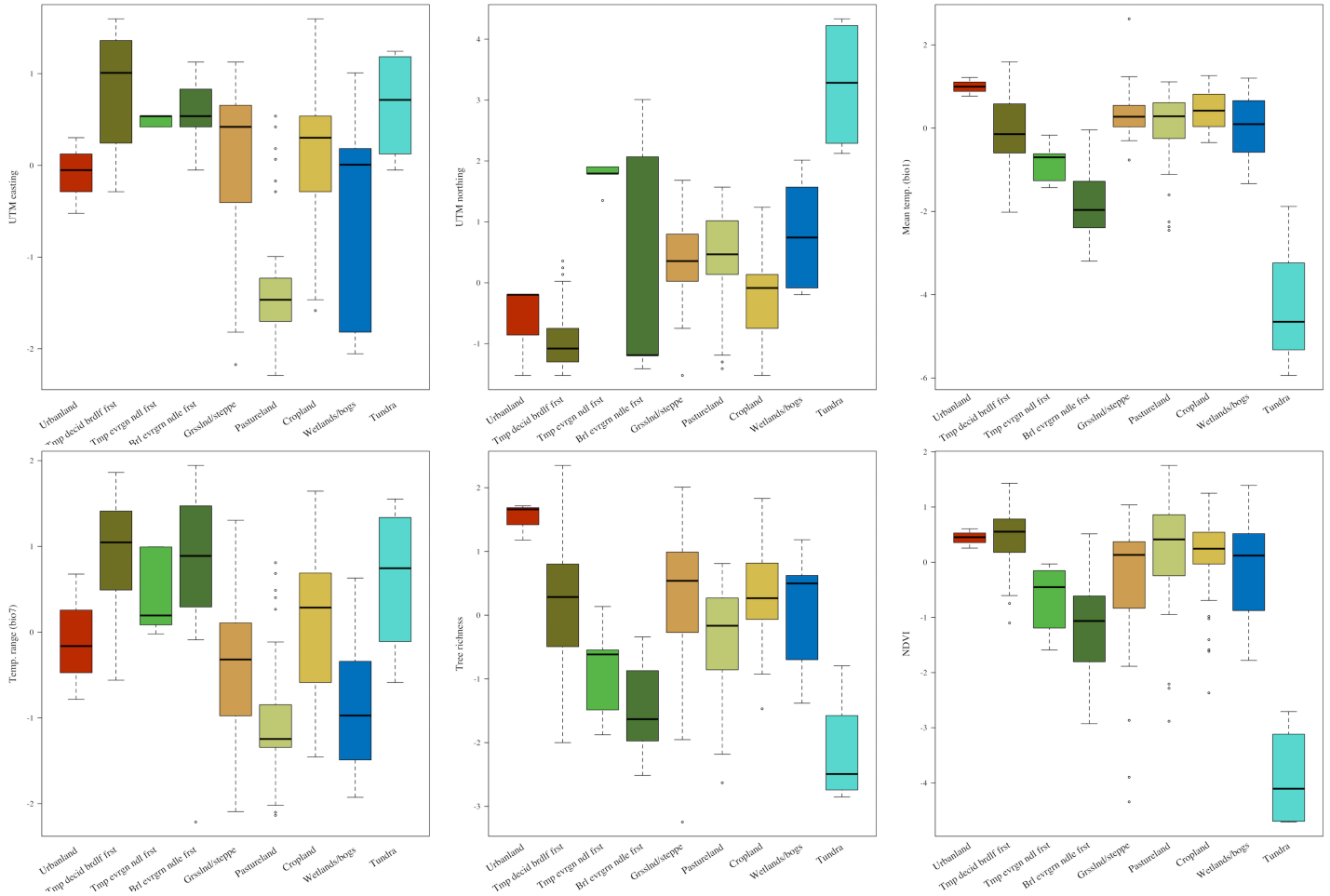

Supplement: Supplementary file 15 — APPENDIX S15. The mean and range in each of the explanatory variables connected to the fruiting records, for the final consensus model for ectomycorrhizal fungi, between each of the land‐use types of the dynamic (ISAM‐HYDE) variable. All variables are scaled. [file APS3-7-e01227-s015.pdf]
